# Trueness, Flexural Strength, and Surface Properties of Various Three-Dimensional (3D) Printed Interim Restorative Materials after Accelerated Aging

**DOI:** 10.3390/polym15143040

**Published:** 2023-07-14

**Authors:** Omar Alageel, Saleh Alhijji, Omar Alsadon, Majed Alsarani, Abdurabu Abdullah Gomawi, Abdulaziz Alhotan

**Affiliations:** 1 Dental Health Department, College of Applied Medical Sciences, King Saud University, Riyadh 12372, Saudi Arabia; smalhijji@ksu.edu.sa (S.A.); oalsadon@ksu.edu.sa (O.A.); malsarani@ksu.edu.sa (M.A.); aalhotan@ksu.edu.sa (A.A.); 2 Dental University Hospital, King Saud University Medical City, Riyadh 11461, Saudi Arabia; agomawi@ksu.edu.sa

**Keywords:** 3D printing, additive manufacturing, restorative dentistry, interim restorations, temporary restorations, aging

## Abstract

Various 3D printing systems for interim fixed dental restorations are commercially available. This study aimed to evaluate the physical and mechanical properties of 3D printed resins used for interim restorations fabricated using various 3D printing systems and printing angulations after accelerated aging. Three different interim restorative materials were provided and printed using their specific 3D printing systems (A: NextDent; B: Asiga; C: Nova3D), and the testing specimens from each system were printed at two building angles: (1) 0° and (2) 90°. The six groups were A1, A2, B1, B2, C1, and C2, with sixteen specimens per group. Half of the specimens in each group (N = 8) were subjected to accelerated aging, including simulated brushing and thermocycling. Three-point bending, surface roughness, and Vickers microhardness tests were performed. Two-way ANOVA and Fisher’s multiple tests were used for statistical analyses. The most accurate systems were found in groups C1 and C2 for length, A1 and B1 for width, and A1 and C1 for height. The specimen trueness only changed after aging for groups B1, B2, and C1. The flexural strength of the A2 group (151 ± 7 MPa) before aging was higher than that of the other groups, and the strength decreased after aging only for groups A1 and A2. The flexural strength, microhardness, and surface roughness of the 3D printed interim resins after aging varied depending on the material, system used, and printing angle.

## 1. Introduction

Provisional, interim, or temporary crowns are an essential part of fixed dental restorations to restore the esthetics and function of intraoral structures and to protect the prepared natural teeth during treatment [1,2,3]. These prostheses can be fabricated either directly on the prepared tooth in the clinic or indirectly in the dental laboratory using an impression or a digital scanner of the patient’s mouth [2,4]. The indirect procedure can be considered a higher-quality, safer, and more convenient alternative for patients than the direct procedure [4]. Interim fixed dental restorations are typically indicated to be used temporarily for a short period before fitting permanent restorations [2,5]. However, the recently improved quality of digitally fabricated interim restorations has made them a viable option for long-term use [4,6].

Digital technologies, such as computer-aided design and computer-aided manufacturing (CAD-CAM) systems, have gained popularity in the fabrication of interim restorations [6]. Digital systems can produce interim restorations using different techniques, such as subtractive (milling) or additive (3D printing) manufacturing methods [6,7,8]. Both digital techniques can produce interim restorations of higher quality and accuracy than those produced using conventional manual techniques. In addition, they exhibit better physical and mechanical properties, including flexural strength and surface properties, than conventionally produced interim crowns [6]. Furthermore, producing a digitally designed restoration in a layer-by-layer pattern using additive or 3D printing technology can be considered a fast and inexpensive approach, leaving less material waste than subtractive methods [8,9]. Recent deployments of 3D printing technologies have shown improved accuracy and reduced cost compared with earlier 3D printing materials and systems, making 3D printed interim restorations increasingly popular in dentistry [9].

Various 3D printing technologies and systems are commercially available for dental applications [8]. Three-dimensional printing technologies include fused deposition modeling (FDM), poly/multi-jetting, powder bed fusion (PBF), stereolithography (SLA), and digital light processing (DLP) [8,10]. DLP is more popular for manufacturing interim fixed crowns because of its superior accuracy, processing time, material wastage, and cost compared to other 3D printing technologies [9,11]. The dental market offers several DLP systems for the 3D printing of interim fixed restorations, such as NextDent (3D systems, Soesterberg, the Netherlands), Asiga (Asiga, Alexandria, Australia), and Nova 3D Master (Nova3D, Shenzhen, China), each offering different features such as speed, resolution, cost, and quality [8].

Polymethyl methacrylate (PMMA) and bis-acrylic resins are frequently used in interim restorations using conventional, milling, and 3D printing methods owing to their adequate physical and mechanical properties and cost-effectiveness [1,3,5,6]. However, self-cured PMMA used in conventional methods has insufficient mechanical properties, whereas enhanced methyl methacrylate (MMA)-based acrylic composite resins used in milling and 3D printing technologies exhibit improved mechanical properties [1,6]. The improvement in the mechanical properties of resin discs and blocks used for milling methods can be due to polymerization with a high degree of conversion and because of the nanofillers added to the photopolymerized resin for 3D printing technology [2]. Interim restoration materials for 3D printing are currently commercially available for SLA and DLP technologies; however, they are limited to FDM, PBF, and poly/multi-jetting technologies [12]. It should be noted that the chemical composition of the 3D printed interim restorative materials has not yet been fully revealed by the manufacturers [8,12].

The accuracy, trueness, and precision of the final printed object can be influenced by the processing parameters and capability of the printer [12,13,14,15]. The mechanical and physical properties of 3D printed objects can also be affected by the materials used and processing techniques applied [11,13,16,17,18]. Several studies have evaluated the properties of interim restoration materials using 3D printing technology, including factors such as accuracy [13,18], surface properties [6,13], and flexural strength [9,10,11,12,14,18,19,20]. However, there have been a limited number of studies that have assessed their performance under accelerated aging conditions, such as thermocycling and brushing, despite the potential long-term use of these restorations [6,20,21]. Thermocycling mimics biological aging of materials by repeatedly exposing dental restorative materials to cycles at various temperatures [6,22]. Artificial brushing tests can help determine the longevity of dental materials by measuring changes in the surface roughness of the evaluated materials [21].

To the best of our knowledge, there is a lack of scientific studies that evaluate and compare the physical and mechanical properties of 3D printed interim restoration materials from different 3D printing systems after accelerated aging. The objective of this study was to evaluate the physical and mechanical properties of interim restoration materials fabricated using various 3D printing systems and printing angulations after accelerated aging. The null hypothesis was that there would be no significant differences in the mechanical and physical properties of the interim restoration materials after accelerated aging using different 3D printing systems. The second null hypothesis was that printing angles of 0° and 90° would present no significant differences in trueness, flexural strength, or surface roughness when producing 3D printed interim fixed restorations.

## 2. Materials and Methods

### 2.1. Specimen Preparation

**Figure 1:**
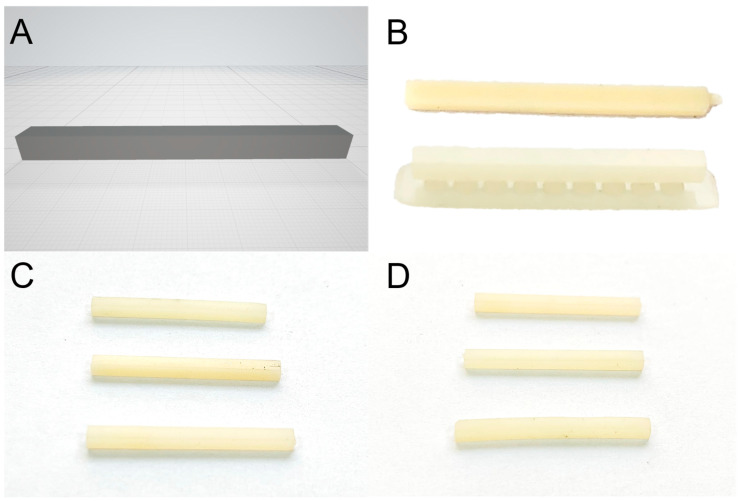
(**A**) STL file used for 3D printing specimens for all groups; (**B**) 3D printed specimens after printing with support at different angulations of 0 (top) and 90 degrees (bottom); (**C**) 3D printed specimens at printing angle of 0° (groups A1, B1, and C1); (**D**) 3D printed specimens at printing angle of 90° (groups A2, B2, and C2).

Six groups of interim crown materials were 3D printed using the digital light processing (DLP) technique, following the manufacturer’s instructions. Three 3D printing systems with their specific materials for interim restorations were selected for this study: (A1 and A2) NextDent 5100 printed with Crown & Bridge NextDent ^®^ (3D Systems, Soesterberg, The Netherlands), (B1 and B2) Asiga MAX printed with Asiga DentaTooth (Asiga, Alexandria, Australia), and (C1 and C2) Nova 3D Master (Nova3D, Shenzhen, China) printed with JamgHe temporary resin (JamgHe, Shenzhen, China). Sixteen rectangular specimens (2 mm × 2 mm × 25 mm) for the three-point bend test according to the ISO10477 standard [6] were printed for each group at building angles of (1) 0° and (2) 90° (Figure 1). The trade names, chemical compositions, and building angles of the products are listed in Table 1.

All testing specimens were designed using open-source CAD software (FreeCAD v.18), saved in the standard stereolithography language (STL) format, and exported to a specific printer to add support and start the production of the testing specimens according to the manufacturer’s instructions. The specimens for all groups were A2 shaded with a printing layer thickness of 50 µm, based on the recommended printing parameters. They were printed at two building angles of 0° and 90°. Subsequently, the supports were removed from the printed object before cleaning with isopropyl alcohol, followed by post-processing polymerization in the post-curing units in line with the manufacturer’s instructions for each system (Figure 1).

**Figure 2:**
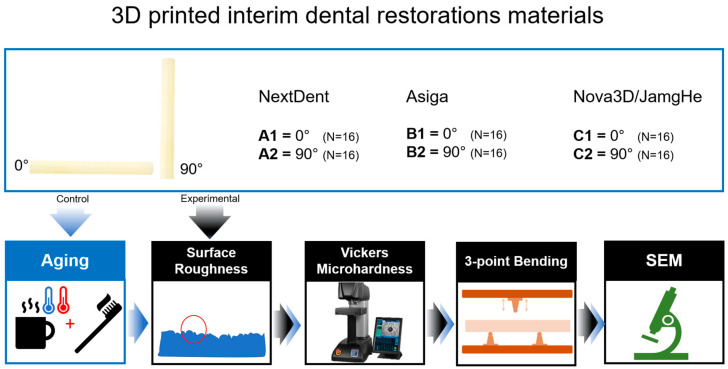
The flowchart of the study process.

Custom-designed holders were designed based on the specimen dimensions and 3D printed (M200 printer and Z-ABS filament; Zortrax SA, Olsztyn, Poland). The holders were created to fix the specimens, provide a secure grip on the specimens to prevent finger injury, and distribute the pressure during the polishing procedure. Similar to the polishing procedure employed in dental laboratories, one side of each specimen was polished under water cooling using a polishing machine (EcoMet/AutoMet 250, Buehler, Lake Bluff, IL, USA) and silicon carbide papers (1000 and 1500 grit), followed by a final polishing with a cloth with a polishing paste (Abraso-Starglanz; Bredent, Senden, Germany). Figure 2 shows a flowchart of the study process.

### 2.2. Accelerated Aging Treatment

Eight specimens from each group underwent accelerated aging to simulate mechanical wear and hydrothermal cycling that occur in the oral environment in accordance with the ISO11505 standard [6]. The accelerated aging procedure included a tooth brushing simulation followed by thermocycling. The first step was to use a tooth brushing simulator (ZM 3; SD Mechatronik GMBH, Feldkirchen Westerham, Germany) equipped with 12 detachable brush heads. In this study, three soft toothbrushes (Oral-B Classic Care 40 M; Procter & Gamble, Surrey, UK) were fixed at brushing stations, where the specimens were fixed horizontally on station holders using 3D printed custom-designed holders. The simulator was set to 27,500 strokes at a brushing speed of 30 s/min and a vertical load of 200 g at a cycling movement of 10 mm, simulating 3 years of brushing [6]. The brushing slurry was prepared by mixing distilled water and toothpaste (Signal; Unilever, London, UK) in a 1:2 ratio and applied every 5000 cycles. After the tooth-brushing simulation, a thermocycler (Huber 1100; SD Mechatronik GmbH, Feldkirchen-Westerham, Germany) was employed by subjecting the specimens to 15-second immersion cycles in cold and hot baths at temperatures of 5 °C and 55 °C, respectively, with a 15-second holding time between the cold and hot baths. To estimate 3 years’ oral consumption, thermocycling was performed for 3500 cycles [6,23].

### 2.3. Trueness Assessment

The dimensions of the test specimens used in the study were measured using an electronic caliper (Fowler High Precision, Newton, MA, USA) to calculate the trueness of 3D printing systems. The dimensions of each test specimen were measured in all three dimensions (width, length, and thickness). Dimensional differences between printed and CAD-designed specimens were calculated.

### 2.4. Surface Roughness Assessment

The surface roughness values of the printed specimens before and after polishing and after accelerated aging for each group were measured using a non-contact optical profilometer (Contour GT; Bruker, Billerica, MA, USA). Five random specimens underwent three separate measurements at various sites, with a threshold of 4%, length of 90 m, and speed of ×2 and VSL measurement type. The mean surface roughness (Sa) values of the 15 measurements were determined in micrometers (μm).

### 2.5. Microhardness Assessment

A Vickers microhardness indenter (Nova 130; Innovatest Europe BV, Maastricht, The Netherlands) was used to measure the microhardness of each group with a 50 g indentation force and a 10-second dwell period. The mean microhardness values of three randomly chosen specimens from each group that had been indented five times at various sites were determined using images taken by a built-in camera at the location of the indentation.

### 2.6. Mechanical Testing

Three-point bending tests were performed using an Instron Universal Testing Machine (Instron Corp., Canton, MA, USA) at a constant speed of 1 mm/min with a 500 N load cell. All specimens (N = 16) from each group were positioned 18 mm apart. Bluehill software (v.2; Instron Corp., Canton, MA, USA) was used to obtain force–deflection curves for each test. Equations (1) and (2) were used to compute the flexural strength (F) and modulus (E), respectively:

F = 3 Fmax L/2 b d2(1)

E = Fy L3/4 δ b d3(2)

where Fmax is the maximum force, L is the distance between the supports, Fy is the yield force, b is the width of the specimen, d is the height of the specimen, and δ is the deflection of the tested specimen [6].

### 2.7. Statistical Analyses

The sample size of the study was determined using G*Power software (v.3.01; Kiel, Germany) according to a pilot study (N = 5) with an estimated effect size of 0.52, alpha of 0.05, and 80% power. Data were examined for normality using a histogram, and the mean and standard deviation (SD) were calculated. The groups were compared and statistically analyzed with a 2-way analysis of variance (ANOVA) and Fisher’s multiple comparison test. The Origin program (v.9.0; Origin Lab, Northampton, MA, USA) was employed for statistical analyses, and the significance level was set at *p* = 0.05.

## 3. Results

### 3.1. Trueness

Table 2 lists the accuracies of the interim restoration materials printed using different 3D printing systems. Discrepancies in trueness for each group were found (*p* < 0.05) in all dimensions in groups A1 and B1, whereas they were only found between length and other dimensions in groups A2, B2, and C2, and no discrepancies in trueness were found in group C1. When assessing the trueness of the same printing system with different building angles, groups A1 and A2 (NextDent system) exhibited significant differences (*p* < 0.05) only in the height dimension. Groups B1 and B2 (Asiga system) showed significant differences (*p* < 0.05) in all dimensions, whereas groups C1 and C2 (Nova system) showed significant differences only in the length dimension. This study identified the most accurate and precise systems for different dimensions, where groups C1 and C2 exhibited the highest length trueness. Similarly, groups A1 and B1 demonstrated superior performance in the width dimension, whereas groups A1 and C1 achieved superior performance in the height dimension. The trueness of the printed specimens remained consistent after the accelerated aging process, except for groups B2 and C1 in the length dimension and group B1 in the width dimension.

### 3.2. Mechanical Testing

**Figure 3:**
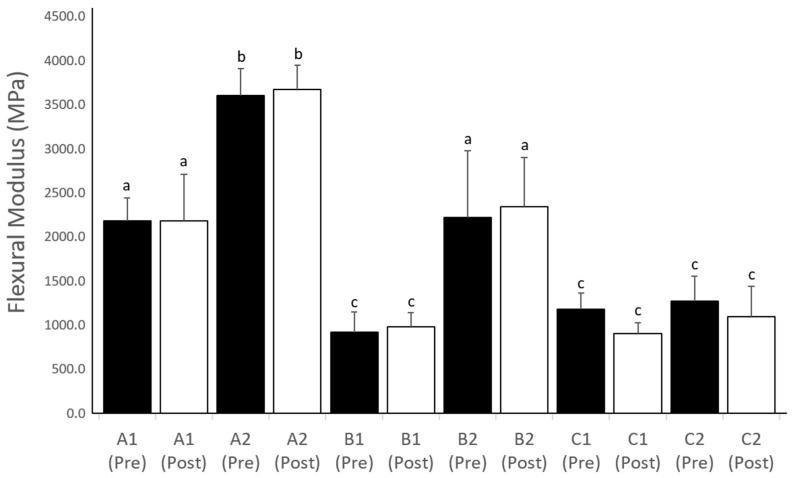
Chart showing the flexural modulus (MPa) of all tested groups before (Pre) and after (Post) aging. The same letter indicates no significant differences between the groups.

**Figure 4:**
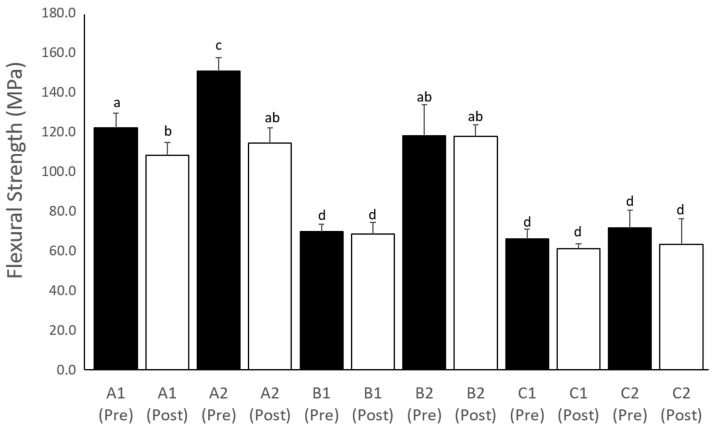
Chart showing the flexural strength (MPa) of all tested groups before (Pre) and after (Post) aging. The same letter indicates no significant differences between the groups.

The mean and standard deviation values of the flexural modulus and flexural strength for all the groups are shown in Figure 3 and Figure 4, respectively. The flexural moduli of A2 were the highest (3606 ± 305 MPa) among the groups, whereas those of groups A1 and B2 (2179 ± 261 and 2223 ± 758 MPa, respectively) were higher (*p* < 0.05) than those of groups B1, C1, and C2 (920 ± 230 MPa, 1180 ± 186 MPa, and 1269 ± 284 MPa, respectively). The flexural moduli of all the groups showed no significant changes (*p* > 0.05) after accelerated aging. In contrast, the flexural strength of the A2 group (151 ± 7 MPa) was higher (*p* < 0.05) than those of the other groups before accelerated aging (pre-aging). Furthermore, the compressive strengths of groups A1, A2, and B2 (108 ± 6, 115 ± 8, and 118 ± 6 MPa, respectively) were significantly stronger (*p* < 0.05) than those of groups B1, C1, and C2 (69 ± 6, 61 ± 3, and 63 ± 13 MPa, respectively) after accelerated aging (post-aging). The flexural strength of all groups did not change (*p* > 0.05) after accelerated aging, except for groups A1 and A2.

### 3.3. Surface Roughness

**Figure 5:**
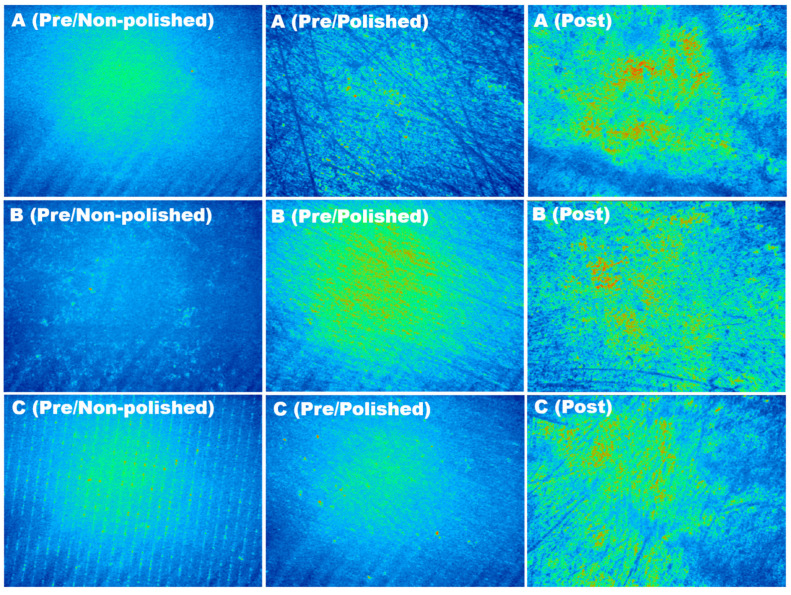
Surface profile images of interim crown materials before (Pre) and after (Post) brushing simulation for groups (**A**): NextDent, (**B**): Asiga, and (**C**): Nova3D. Color variation corresponds to the surface depth.

The mean surface roughness and standard deviation (Sa ± SD) of all 3D printed interim crown materials before accelerated aging (pre-aging) with non-polished and polished surfaces, as well as after accelerated aging (post-aging), are presented in Table 3. The mean Sa values of groups A and C (A1, A2, C1, and C2) (from 0.211 to 0.227 µm) were significantly (*p* < 0.05) lower than those of the B groups (B1 and B2) (0.234 ± 0.012 and 0.252 ± 0.048 µm, respectively) before aging and any polishing process. However, the mean Sa values of group A (A1 and A2) were significantly (*p* < 0.05) lower (0.213 ± 0.001 and 0.208 ± 0.011 µm, respectively) than those of the other groups (from 0.220 to 0.234 µm) after accelerated aging. Only groups A1, B2, and C2 exhibited reduced Sa values (*p* < 0.05) after accelerated aging. Surface profile images of one group from each 3D printing system are shown in Figure 5.

### 3.4. Microhardness

**Figure 6:**
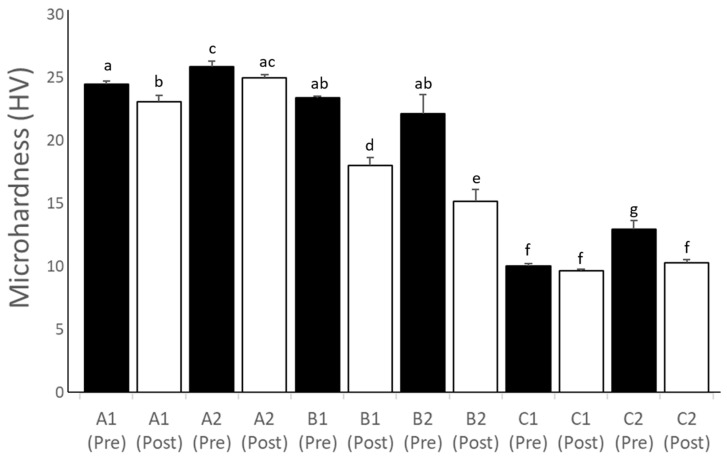
Chart showing the microhardness values (HVs) of the interim crown materials before (Pre) and after (Post) accelerated aging. The same letter indicates no significant differences between the groups.

Figure 6 shows the results of the microhardness values (HVs) of the interim crown materials before (pre-aging) and after (post-aging) accelerated aging for all groups. All groups showed reduced microhardness values (*p* < 0.05) after accelerated aging, except for C1. Before accelerated aging, the highest microhardness values (*p* < 0.05) were observed in group A2 (25.9 HV), followed by groups A1, B1, and B2 (24.5, 23.4, and 22.1 HV, respectively) and C2 (12.9 HV) and C1 (10.0 HV). After accelerated aging, group A2 (25.0 HV) had the highest microhardness values, followed by groups A1 (23.0 HV), B2 (22.1 HV), and B1 (18.0 HV). The lowest microhardness values after accelerated aging were observed (*p* < 0.05) for C2 (10.3 HV) and C1 (9.6 HV).

## 4. Discussion

This in vitro study aimed to assess the impact of accelerated aging on the flexural strength, surface roughness, and microhardness of interim crown materials fabricated using various 3D printing systems and two building angulations. It was hypothesized that there would be no significant difference in the mechanical and physical properties of interim crown materials printed using different 3D printing systems and printing angles of 0° and 90° after accelerated aging. Therefore, both null hypotheses were rejected. The outcomes of this study are clinically relevant because it explains the longevity of interim restoration materials taking into account that periodontal health and plaque accumulation are greatly influenced by the surface roughness and hardness of restoration materials [6,23,24]. In fact, an accurate and high marginal fit of interim fixed restorations with satisfactory mechanical and physical properties can withstand long-term use [6,7,18,19,22].

The results of this study demonstrated that the trueness of 3D printed interim fixed restorations is influenced by the type of printing system, printing angulation, and the aging process. It can be observed that a printing direction of 0° provides more accurate height dimensions for all groups and lengths for the B1 (Asiga) and C1 (Nova3D) systems. Previous studies have also suggested that printing with a print angulation of less than 30° results in superior trueness compared to printing with a larger printing angle; printing with a 90° angle is the least accurate and precise [25,26]. Accelerated aging affects the specimen dimensions, particularly for groups B2 and C1 in the length dimension and group B1 in the width dimension. These outcomes could be attributed to shrinkage effects resulting from the heat and cold cycles during thermocycling as well as water absorption by the printed resin [15,21]. Groups A1 and A2 (NextDent) did not undergo significant changes after accelerated aging, potentially due to their microstructure, which also had less effect on microhardness following accelerated aging.

The highest flexural strength among all the tested groups was found in the A2 group (NextDent printed at 90°), followed by B2 (Asiga printed at 90°), and A1 (NextDent printed at 0°). This study found that printing at 90° resulted in greater strength for groups A and B (NextDent and Asiga systems, respectively) but not for group C (Nova3D system). These findings contrast with previous studies suggesting that a building direction of 90° exhibits the lowest flexural strength, with printing at 30° showing a higher flexural strength than at 0° and 90° [9]. Another study highlighted that the flexural strength of 3D printed specimens can vary depending on the loading direction of the three-point bending test and the growing direction of the 3D printed specimens [14,27,28]. Specifically, when the loading direction is parallel to the growing direction, the specimens printed at 0° exhibit greater flexural strength than those printed at 90°, but the difference is not significant when the loading direction is perpendicular to the growing direction [14]. In this study, the flexural test was applied in loading directions perpendicular to the growth direction of the specimens, which could explain the observed results. When a force is applied, interlayer bonding in the load direction causes separation between the layers [28]. Thus, the flexural strength of 3D printed interim fixed restorations is influenced by the printing angle but also depends on the specific 3D printing system employed. Furthermore, the mechanical properties of the interim material are affected by factors such as the chemical composition, molecular structure, and filler content of the 3D printing resin, as reported in previous studies [3,29].

The flexural strength of the interim crown materials was reduced after accelerated aging, specifically in groups A1 and A2 (NextDent printed at 90° and 0°, respectively). However, even after aging, the flexural strengths of A1 and A2 remained comparable to those of B2 and higher than those of other groups (B1, C1, and C2). Furthermore, groups A1, A2, and B2 exhibited significantly higher flexural strengths than other groups after accelerated aging. The decrease in flexural strength of groups A1 and A2 after accelerated aging may be attributed to the weakening and degradation of the resin matrix [6,15,23]. Nevertheless, the flexural strength values for all tested groups of interim fixed restoration materials met or exceeded the minimum ISO10477 standard for interim fixed restoration at a flexural strength of 50 MPa [19]. The flexural moduli of all the groups remained unchanged after accelerated aging. Groups A and B demonstrated significantly higher microhardness values than group C, which could explain the higher flexural strength observed in groups A and B than that in group C.

The highest surface roughness (Sa) value found in this study was for the B2 group both before and after the accelerated aging process. This might be due to the printing technology and material used and the curing light used for polymerization [16]. In addition, build angulation, layer thickness, and position on the build platform can influence surface roughness [30]. However, the specimens used in this study followed manufacturers’ recommendations to minimize manufacturing discrepancies [6,30]. The surface roughness values (Sa) obtained in this study are in agreement with those obtained in a previous study [30]. Only groups A1, B2, and C2 were influenced by the accelerated aging process because of the brushing effects of the tooth-brushing simulation and heat of thermocycling [6,21,22]. The lack of a significant effect might be related to their microstructure compared with the other groups. The printing angle plays a significant role in surface roughness because 3D printing builds up the object in a layer-by-layer pattern, and the junction between the layers can result in surface roughening, as shown in Figure 5C.

Thermocycling alters the physical characteristics of the resin by allowing water molecules to permeate the resin, causing resin expansion and breakdown of the polymeric matrix, which may contribute to the decrease in microhardness after thermocycling [6,23]. Since the microhardness values of all groups were lower than the tooth enamel hardness, they are considered for use in dental restorations as they are non-abrasive to natural teeth [13].

The interim resins used in this study for each group were selected using the same manufacturer as the 3D printer and the manufacturers’ instructions were carefully followed to reduce manufacturing inconsistencies. Previous studies have assessed the effects of various printing parameters on the mechanical properties of 3D printed objects [26,27,28,30]. In addition, the chemical composition of interim materials can influence the mechanical properties and surface roughness of 3D printed objects [3,30]. A previous study found that filler content and increased polymerization can improve mechanical strength [3]. Additionally, it was found that filler particle size is linked to increased surface roughness [3,22,28]. The interim resin materials used in each 3D printing system were different, which can explain the variation in the mechanical properties and surface roughness between the 3D printed objects.

This study had some limitations due to the fact that it was conducted in in vitro conditions using flat specimens that may not accurately reflect in vivo conditions. Further research is required to evaluate the color stability, microbial adherence, and mechanical properties of different 3D printing systems. Additionally, the study was limited to a small number of printing materials, building directions, and aging techniques. Future investigations could compare printing resin materials and procedures by considering the polymerization time and printing methods. Further research is needed to better understand the effects of filler content and polymerization on the mechanical properties and surface roughness of 3D printed objects. Finally, microflexural testing is a more suitable option for testing dental restoration materials and has more clinical relevance because dental restorations are small [31].

## 5. Conclusions

The aging process, including thermocycling and brushing simulation, affected the trueness, flexural strength, microhardness, and surface roughness of the tested 3D printed interim restorations, depending on the 3D printing system used and the printing angle. The 3D printed interim resins printed at 0° exhibited better dimensional trueness and lower strength than those printed at 90°.

## Figures and Tables

**Table 1:** Three-dimensional printing systems and chemical composition of interim crown materials of all groups provided by the manufacturers.

Group	Resin	Chemical Composition	Printing System
A1 and A2	Crown & Bridge NextDent ^®^; Nextdent, Soesterburg, the Netherlands	Methacrylic oligomers, methacrylate monomer, phosphine oxides, pigment	NextDent 5100; Nextdent, Soesterburg, the Netherlands
B1 and B2	DentaTooth; Asiga, Alexandria, NSW, Australia	Trimethyl-4,13-dioxo3,15-dioxa-5,12-diazahexadecane-1,16-diyl bismethacrylate, tetrahydrofurfuryl methacrylate, and diphenyl phosphine oxide	Asiga MAX; Asiga, Alexandria, Australia
C1 and C2	JamgHe temporary resin; JamgHe, Shenzhen, China	NP	Nova 3D Master; Nova3D, Shenzhen, China

NP: Not Provided.

**Table 2:** Mean deviation errors (µm) of each printed specimen in comparison to the original STL.

	Pre-Aging (Mean Error in µm ± SD)			Post-Aging (Mean Error in µm ± SD)		
Group	Length (L)	Width (W)	Height (H)	Length (L)	Width (W)	Height (H)
A1	190 (40) ^A,a^	40 (20) ^A,b^	−10 (10) ^A,c^	220 (50) ^A,a^	30 (20) ^A,b^	−10 (2) ^A,c^
A2	220 (40) ^A,a^	70 (40) ^A,b^	50 (10) ^B,b^	220 (40) ^A,a^	70 (30) ^A,b^	50 (10) ^B,b^
B1	70 (30) ^B,a^	100 (20) ^B,b^	−70 (20) ^C,c^	90 (40) ^B,a^	40 (10) ^A,d^	−50 (20) ^C,c^
B2	−130 (30) ^C,a^	50 (10) ^AC,b^	60 (20) ^B,b^	−70 (30) ^C,d^	40 (10) ^A,b^	50 (10) ^B,b^
C1	−30 (20) ^D,a^	−70 (40) ^D,a^	−30 (30) ^AD,a^	10 (30) ^D,b^	−90 (20) ^B,a^	−10 (10) ^A,a^
C2	30 (20) ^BE,a^	−70 (40) ^D,b^	−60 (40) ^CE,b^	50 (30) ^BE,a^	−60 (20) ^C,b^	−90 (20) ^D,b^

Different uppercase superscript letters indicate significant differences between the groups in the columns (*p* < 0.05). Different lowercase superscript letters indicate significant differences between the groups in the rows (*p* < 0.05).

**Table 3:** Surface roughness (Sa) of the interim crown materials before (pre-aging) treatment with non-polished and polished surfaces and after (post-aging) for all groups.

Group	Pre-Aging (Non-Polished)	Pre-Aging (Polished)	Post-Aging (Polished)
A1	0.226 (0.002) ^A,a^	0.224 (0.003) ^A,a^	0.213 (0.001) ^A,b^
A2	0.227 (0.002) ^A,a^	0.213 (0.022) ^B,b^	0.208 (0.011) ^A,b^
B1	0.234 (0.012) ^B,a^	0.219 (0.006) ^C,b^	0.220 (0.006) ^B,b^
B2	0.252 (0.048) ^B,a^	0.217 (0.002) ^C,b^	0.234 (0.003) ^C,c^
C1	0.211 (0.003) ^A,a^	0.225 (0.004) ^A,b^	0.229 (0.002) ^D,b^
C2	0.224 (0.003) ^A,a^	0.190 (0.004) ^D,b^	0.226 (0.001) ^D,a^

Different uppercase superscript letters indicate significant differences between the groups in the columns (*p* < 0.05). Different lowercase superscript letters indicate significant differences between the groups in the rows (*p* < 0.05).

## Data Availability

Data of the study are available on request from the corresponding author.
